# On the Doubtful Origin of Cow-Pock Matter

**Published:** 1806-06

**Authors:** Robert Kinglake

**Affiliations:** Taunton


### On the doubtful Origin of Cow-pock Matter, by Dr. Kinglake.

*To the Editors of the Medical and Physical Journal.*

Gentlemen,

It has been insisted on with more or less positiveness by different inquirers, that the source of Cow-pock Matter is a diseased secretion in the heels of the horse, usually denominated *grease*. The advocates of this opinion concur in asserting, that this equine fluid or *grease* applied to the cow's teat, will, under suitable circumstances, produce the genuine pock, which promises soon to supersede inoculation for the small-pox, and eventually to exterminate that severe disease altogether. It is not my design to question the permanent efficacy of cow-pox in preventing small-pox; but to object to the unqualified terms in which this benign disease is ascribed to what must yet be considered as a doubtful source.

Mr. Ring has laboured to establish the probable advantages of vaccine over variolous inoculation much more successfully than he can be allowed to have elucidated the identity of vaccine and equine matter, which he asserts, under either appellation, equally destroys the innate susceptibility for the small-pox infection.

Mr. Ring thus expresses himself, in page 168 of the 84th Number of your Journal, "That the disease called the genuine cow-pox proceeds from the horse is as clearly proved as any other fact in medical science; a similar disease, endowed with similar prophylactic virtue, having been repeatedly produced in the human subject, without the intervention of the cow. This, among other instances, has been done by Dr. Sacco, of Milan, who was before sceptical on the subject, in the presence of several medical men assembled for that purpose, one of whom was a friend of Dr. De Carro. Matter of this kind has been transmitted to Dr. De Carro, and to Dr. Friese, of Silesia, and has also succeeded in their hands. They now inoculate indiscriminately with equine or vaccine matter, and with equal effect. "One of our Medical Journals, some months ago," continues Mr. Ring, "announced that another practitioner in Germany had also succeeded in an experiment with equine matter."

 In

In my view of the possible hazard of trying what might be the event of inserting the acrid and fœtid matter issuing from the diseased heels of the horse; it would not be warrantable for me directly to extend the experiment to the human subject. Mr. Ring has, indeed, adduced some authorities for the practice; but surely, if it be so indisputably safe and efficacious as he represents it to be, how is it that not a solitary experiment on the subject has been made in our own country? in the very country where the idea of the supposed identity originated? Perhaps others, as well as myself, have been feelingly deterred from risking the precarious consequences of introducing into the human animal economy a foul secretion from a distempered part of a quadruped; but this humane disinclination should not have restrained a fair trial of its efficacy on the cow, for the purpose of ascertaining the validity of the opinion, that contends for the cow-pock disease deriving its origin from the horse, and that it is imparted to the cow by the hands of the milkers, contaminated with the infectious matter alluded to. If this be a fact, occurring in the occasional manner here stated, the same effect certainly ought to be produced by directly inoculating the cow's teat with the matter afforded by the horse's heel suffering under the disease called *grease*. Has this ever been put to the test of experiment, and with what result? It has appeared to me, that an inquiry, so easily practicable, should not be a subject of conjecture, but of positive examination. With a view to obtaining this demonstrative proof, matter was lately procured for me from a horse severely afflicted with the disease called *grease*. It was taken at the limpid and more purulent stages of the distemper. Distinct dossils of lint, dripping with each sort of this fluid, were inserted by me into different teats of the same heifer, by means of an horizontal incision made with a seton needle. The apertures were afterwards bound round with tape, for the purpose of retaining during a few days on the sore surfaces, the full efficacy of the application. On the third day the bandages were removed, when the incisions exhibited a slight redness, without tension; on the seventh day, a dry cicatrix evinced the complete inefficacy of the matter which had been inserted. On the the same day, the operation was renewed in a similar manner on two other teats of the same heifer, and precisely with the like consequences. No effect approaching to suppurative inflammation ensued, and certainly not a higher degree of excitement was produced than would necessarily necessarily have followed an equal incision made with a clean instrument.

This is an extraordinary fact; but it either proves the inoffensive properties of the matter in question, or the difficulty of exciting local inflammation in an animal like the cow, fortified by the balmy health of uncorrupted nature. On inquiring particularly of the farrier, who procured the matter for the experiments here detailed, and who professed to have had much experience in the disease which furnished it, whether it had ever inconvenienced him by irritating any open sore which he might have had, and with which it might have come in contact in the course of his dressing the distempered parts? or whether he had ever known it to induce eruptions, either on himself, or others? he answered, that he was unconscious of any such effect from it, and that he had always considered it perfectly harmless.

A man servant officiating as carter to a farmer, and who assisted me in the above experiments, also declared that he had long been in the habit of managing *greasy* heeled horses, and afterwards of milking cows, but that neither any eruption on himself, nor on the teats of the cows he had milked, had to his knowledge ever ensued.

It may be objected, that my experiments on this subject have not been sufficiently numerous to be decisive. Perhaps they have not; but surely they go far enough to shake the absolute assertion of Mr. Ring, respecting the identity of equine and vaccine pock matter. In my own judgment, indeed, they are conclusive; and unless Mr. Ring, or some other advocate for the opinion that the cow-pock disease originates from the *grease* of horses heels, can impart the distemper to the cow by inoculating the animal with the said matter; it is but just to consider their assertion on the subject as gratuitous, and of course inadmissible.

Were Mr. Ring's notion, that the source of the vaccine disease is in the distempered heels of the horse, well founded, it would be injudicious to extend it to the human subject through the intermedium of the cow. By this procedure, its native qualities may be impaired, and perhaps its uniform efficacy in preventing the small-pox may be lessened.

If the diseased heels of the horse be the genuine fountain of the vaccine pock, an ample supply of infallible matter may be there obtained. Crowded stables will generally be its reservoirs. This anti-variolous agent, indeed, will abound in the proportion in which horses are exces cessively fed, insufficiently exercised, and negligently groomed.

The inquiry, however, is purely experimental; the discovery of truth is worthy of the best efforts of philosophy; nor should either prejudice or intemperate zeal interfere with a dispassionate endeavour to solve the question at issue. If Mr. Ring can warrant his confident persuasion of vaccine pock being the offspring of an equine secretion, by the experiment of inoculating either the cow or the human subject with that fluid, and thereby producing the vaccine disease, he shall have my grateful assent to the important fact; but until then, both my reasoning and experience on the subject, will oblige me to consider the two diseases as radically distinct, as the animals are in which they occur.

The remarks of your Correspondent, Mr. Namlat, in pages 219—20, of your Journal for the preceding month, respecting my opinion of the identity of the vaccine and variolous diseases breathe too much the spirit of petulant invective and offensive reproach, to merit any reply. If that gentleman would promote the interest of truth, he should attempt it by liberal investigation, not by disingenuous and inapposite reflections,

I am, &c.

*Taunton, April* 9, 1806. ROBERT KINGLAKE.

